# Galectin-3 Plasma Levels Are Associated with Risk Profiles in Pulmonary Arterial Hypertension

**DOI:** 10.3390/diagnostics10110857

**Published:** 2020-10-22

**Authors:** Laura Scelsi, Stefano Ghio, Benedetta Matrone, Letizia Mannucci, Catherine Klersy, Serenella Valaperta, Annalisa Turco, Alessandra Greco, Giuseppe Derosa, Luigi Oltrona Visconti

**Affiliations:** 1 Division of Cardiology, Fondazione IRCCS Policlinico S.Matteo, 27100 Pavia, Italy; l.scelsi@smatteo.pv.it (L.S.); benedetta.matrone@gmail.com (B.M.); letizia.mannucci@gmail.com (L.M.); a.turco@smatteo.pv.it (A.T.); grecale1985@gmail.com (A.G.); l.oltronavisconti@smatteo.pv.it (L.O.V.); 2 Biometry and Clinical Epidemiology, Fondazione IRCCS Policlinico S.Matteo, 27100 Pavia, Italy; klersy@smatteo.pv.it; 3 Clinical Chemistry, Fondazione IRCCS Policlinico S.Matteo, 27100 Pavia, Italy; s.valaperta@smatteo.pv.it; 4 Dipartimento di Medicina Interna e Terapia Medica Università di Pavia, Fondazione IRCCS Policlinico S.Matteo, 27100 Pavia, Italy; g.derosa@smatteo.pv.it

**Keywords:** pulmonary arterial hypertension, galectin-3, prognosis

## Abstract

Galectin-3 is a circulating biomarker of fibrosis whose prognostic role in pulmonary arterial hypertension (PAH) has not been fully explored. We undertook a pilot study to evaluate the relationship between galectin-3 plasma levels and validated risk scores in PAH. The study included 70 PAH patients admitted to a single referral center from June 2016 to June 2018. Patients were stratified according to the REVEAL 2.0 risk score, according to the parameters suggested by the European Society of Cardiology and European Respiratory Society (ESC/ERS) Guidelines, and according to a focused echocardiographic assessment of right heart performance. The association between galectin-3 levels and risk profiles was evaluated by generalized linear regression model with adjustment for etiology. Galectin-3 plasma levels increased linearly in the three risk strata based on the REVEAL 2.0 score (from 16.0 ± 5.7 in low-risk to 22.4 ± 6.3 in intermediate-risk and in 26.9 ± 7.7 ng/mL in high-risk patients (*p* for trend < 0.001). Galectin-3 levels were significantly lower in low-risk patients defined according to the prognostic parameters of ESC/ERS Guidelines (delta between low-risk and intermediate/high-risk = −9.3, 95% CI −12.8 to −5.8, *p* < 0.001, *p* < 0.001). Additionally, galectin-3 levels were lower in the low-risk profile defined on the basis of the echocardiographic evaluation of right heart performance (delta between low-risk and intermediate-/high-risk = −6.3, 95% CI −9.9 to −2.7, *p* = 0.001). Galectin-3 plasma levels are directly associated with several risk profiles in PAH patients. The prognostic role of this biomarker in PAH is worthwhile to be explored in larger prospective studies.

## 1. Introduction

Although a variety of biomarkers have been explored and associated with prognosis in pulmonary arterial hypertension (PAH), current International Guidelines only recommend the evaluation of brain natriuretic peptide (BNP) or the N-terminal fragment of pro-BNP (NT-pro-BNP) at diagnosis and for longitudinal follow up of patients, since these markers correlate with the extent of myocardial dysfunction [1].

Circulating biomarkers of fibrosis are of high potential interest. In fact, as much as in heart failure, right ventricular remodeling in PAH involves not only the cardiomyocytes, but also non-myocyte cells and the extracellular matrix [2,3]. Galectin-3 (Gal-3), a β-galactoside-binding lectin, is a promoter of inflammation and fibrosis [4]. This biomarker has been correlated with prognosis in heart failure with reduced or preserved ejection fraction [5,6]. As a result, it has been approved by the US Food and Drug Administration as a new biomarker for additive risk stratification in heart failure (Class IIb recommendation in American Heart Association/American College of Cardiology Guidelines) [7]. Elevated Gal-3 concentrations are also associated with interstitial lung abnormalities coupled with a restrictive pattern, suggesting a potential role for Gal-3 in early stages of pulmonary fibrosis [8].

Insights into the prognostic role of Gal-3 in pulmonary arterial hypertension are lacking.

We therefore undertook a pilot study with the aim to evaluate the relationship between Gal-3 plasma levels and several validated risk profiles in PAH patients. First, we assessed Gal-3 levels in three risk strata (low-, intermediate-, and high-risk) defined according to the REVEAL 2.0 risk score. The Registry to Evaluate Early and Long-term PAH Disease Management (REVEAL), initiated in US in 2006, is so far the largest registry of patients with PAH and analysis of its data has enabled development of a risk score calculator to predict 1-year survival in such patients, which has recently been updated [9,10]. Second, we assessed Gal-3 levels using a stratification tool based on the clinical, hemodynamic, echocardiographic, and functional data recommended by European Society of Cardiology/European Respiratory Society (ESC/ERS) PAH Guidelines [11]. Finally, Gal-3 levels were also evaluated on the basis of a focused echocardiographic evaluation of right heart performance [12,13].

## 2. Materials and Methods

### 2.1. Patients

The study includes 70 PAH patients diagnosed according to Guidelines recommendations [1], aged more than 18 years, admitted to a single referral center for PAH in Italy from September 2016 to June 2018. There were 15 incident cases, in whom clinical and hemodynamic data were collected at the moment of the diagnosis, and 55 prevalent cases, hospitalized to repeat right heart catheterization and to escalate targeted therapy. Exclusion criteria were chronic renal damage with glomerular filtration rate lower than 30 mL/min. WHO functional class assessment, 6 min walking test and echocardiography were performed within 24 h from the hemodynamic evaluation.

The investigation conforms to the principles outlined in the Declaration of Helsinki. The Ethical Committee gave the approval for the analysis (Ethical Committee of IRCCS Policlinico S.Matteo Hospital, Pavia, prot.44784/2011, date of approval: 15 December 2011). All patients signed an informed consent agreement approved by the Institutional Review Board of Fondazione IRCCS Policlinico S. Matteo for observational, non-pharmacological, non-sponsored studies, which complies with the Italian legislation on the privacy (Codex on the Privacy, D. Lgs. 30 giugno 2003, n. 196).

### 2.2. Blood Samples and Analysis

Whole blood samples were collected through peripheral venipuncture in fasting subjects and analyzed for plasma BNP. Samples for the evaluation of Gal-3 were collected, centrifugated (4000 g × 10 min) and frozen (−20 °C) and then analyzed in one session with an automated test that quantitatively measures galectin-3 in human serum or plasma using the ELFA (Enzyme-Linked Fluorescent Assay) technique (Biomérieux Clinical Diagnostic, Firenze, Italia).

### 2.3. Echocardiographic Examination

A standard M-mode, two-dimensional, and Doppler study was performed using a commercially available equipment (Vivid 9, GE Healthcare, Little Chalfont, UK). The examination included the following parameters: right ventricular (RV) end-diastolic diameter (RVD); tricuspid annular plane excursion (TAPSE); RV areas and fractional area change (FAC); right atrial area (RAA); end-diastolic eccentricity index of the left ventricle (LVEI-d); systolic pulmonary artery pressure (PAP); degree of tricuspid regurgitation; right atrial pressure (RAP) estimated on the basis of inferior vena cava diameter and its respiratory variation.

### 2.4. Six-Minute Walk Test

A non-encouraged test was performed according to the American Thoracic Society guidelines. Total distance walk (meters) and oxygen saturation were recorded.

### 2.5. Right Heart Catheterization

Right heart catheterization was performed using a balloon-tipped catheter. The following hemodynamic parameters were measured or calculated: pulmonary artery wedge pressure (PAWP); systolic, diastolic and mean PAP; RAP; cardiac output (CO), calculated by thermodilution or by the Fick method in case of severe tricuspid regurgitation; cardiac index (CI); pulmonary vascular resistances (PVR); pulmonary arterial compliance (PCa) estimated dividing the blood volume driven from each heartbeat in the pulmonary vascular tree, namely the stroke volume (SV), by the corresponding change in pulmonary artery pressure, i.e., pulse pressure (PP): PCa ≈ SV/PP (mL/mmHg); systolic, diastolic and mean systemic blood pressure.

### 2.6. Clinical Evaluation

The world health organization (WHO) functional class was assessed as well as the presence of signs of right ventricular dysfunction (peripheral swelling, ascites, hepatomegaly, jugular turgor, hepato-jugular reflux) and the occurrence of recent syncope.

### 2.7. Risk Stratification

Patients were stratified according to the REVEAL 2.0 risk score calculator and the three-category risk score was applied: low risk was defined as a score of ≤6, intermediate risk was defined as a score of 7 or 8, and high risk was defined as a score of ≥9 [10]. Patients were also categorized according to the presence of four low-risk parameters (WHO functional class I or II, 6-min walking distance (6MWD) > 440 m, RAP < 8 mmHg and CI ≥ 2.5 L/min/m^2^) included in a simplified tool based on the ESC/ERS Guidelines [11]; patients with four or three low-risk criteria were considered low-risk patients, patients with two or one low-risk criteria were considered intermediate-risk patients and patients with no low-risk criteria were considered high-risk patients. Additionally, patients were categorized according to a stratification tool based on three echocardiographic parameters of right heart performance: TAPSE (low-risk threshold > 15 mm), degree of tricuspid valve regurgitation (low-risk threshold mild or none), LVEI-d (low-risk threshold < 1.7). Patients were categorized as high-risk in case of TAPSE ≤ 15 mm and LVEI-d ≥ 1.7, low-risk if they had TAPSE > 15 mm and none or trivial tricuspid regurgitation, and intermediate-risk in other cases [12,13].

### 2.8. Statistical Analysis

For categorical variables, absolute and relative (%) frequencies were computed; for continuous variables, mean and standard deviation (SD) or median and 25–75 th percentiles were reported. The clinical features of the patients with low/high Gal-3 plasma levels were compared by the Student t test or the Mann-Whitney U test (continuous data) or the exact Fisher test (categorical data). The test for trend was used to assess the monotonic association of risk score and Gal-3 at univariable analysis. The association of Gal-3 plasma levels and risk profiles was evaluated by generalized linear regression model, with adjustment for etiology since patients with systemic sclerosis have higher plasma levels of Gal-3. The adjusted difference in the plasma level of Gal-3 between categories of risk was derived from the model; 95% confidence intervals (95% CI) were computed. The likelihood ratio test was used to assess linearity of effect. Stata 15.1 (Stata Corp, College Station, Texas, USA) was used for computation. A two-sided *p* < 0.05 was retained for statistical significance.

## 3. Results

### 3.1. Clinical Characteristics

There were 15 incident patients and 55 prevalent patients. Mean age was 56 ± 16 years. Most of the patients were women (*n* = 48, 68%), in WHO functional Class II (*n* = 42, 60%) and most had idiopathic/heritable/anorexigen PAH (*n* = 37, 52%) or PAH associated with systemic sclerosis (SSc) (*n* = 17, 24%). Gal-3 mean plasma levels in the entire population were 20.4 ± 7.8 ng/mL. Patients with SSc had higher levels of Gal-3 as compared to idiopathic/heritable/anorexigen PAH (respectively: 24.0 ± 8.2 ng/mL vs. 17.9 ± 7.4 ng/mL, *p* = 0.005).

Table 1 shows the clinical, hemodynamic, and echocardiographic characteristics of the study population according to Gal-3 plasma levels (i.e., below or above the median value of 19.3 ng/mL). Higher Gal-3 levels were associated with poorer clinical conditions, more advanced hemodynamic profile, greater RV dysfunction at echocardiography.

### 3.2. Galectin-3 Levels and Risk Profiles According to REVEAL 2.0 Risk Calculator

**Figure 1:**
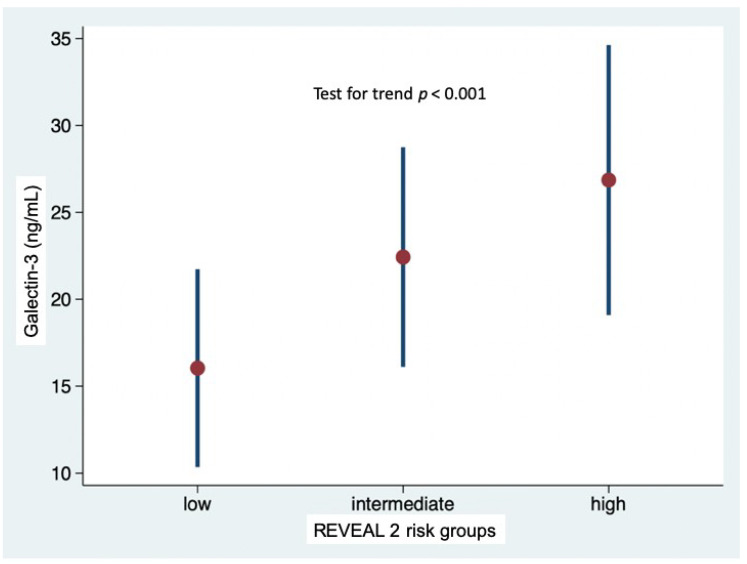
Galectin-3 plasma levels in the low-, intermediate- and high-risk strata defined according to REVEAL 2.0 risk score calculator.

**Figure 2:**
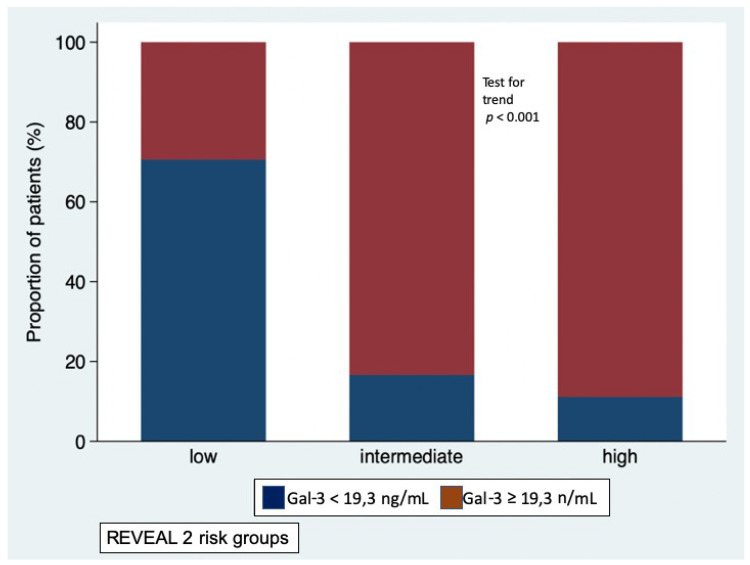
Proportion of patients with Galectin-3 plasma levels below the median value (blue bars) and above the media value (red bars) in the low-, intermediate-, and high-risk strata defined according to REVEAL 2.0 risk score calculator.

At baseline, the majority of patients (*n* = 34, 49%) were in the low-risk stratum, 18 were in the intermediate-risk stratum and 18 were in the high-risk stratum. At generalized linear regression, the REVEAL 2.0 score was significantly associated with Gal-3 levels (*p* < 0.001), while adjusting for etiology. Gal-3 levels increased linearly with the increase in risk (likelihood ratio test for linearity *p* = 0.589); Gal-3 levels were 16.0 ± 5.7 in low-risk stratum, 22.4 ± 6.3 in intermediate-risk stratum, and 26.9 ± 7.7 ng/mL in high-risk stratum (*p* = 0.002 low-risk vs. intermediate-risk; *p* < 0.001 low-risk vs. high-risk; *p* = 0.189 intermediate-risk vs. high-risk) (Figure 1). No interaction was observed with etiology of PAH (i.e., idiopathic or associated with systemic sclerosis, *p* for interaction = 0.364). As shown in Figure 2, the proportion of patients with Gal-3 plasma levels below or above the median value of 19,3 ng/mL was different in the low-risk, intermediate-risk and high-risk strata (test for trend *p* < 0.001).

### 3.3. Galectin-3 Levels and Risk Profiles According to ESC/ERS Guidelines Criteria

**Figure 3:**
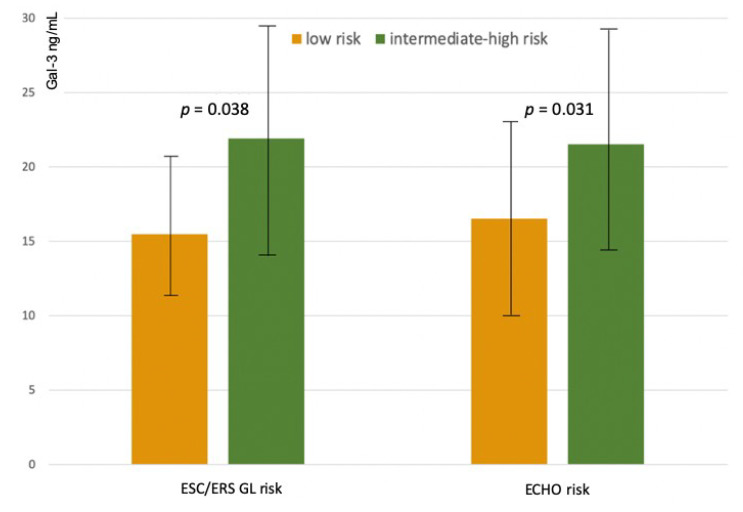
Galectin-3 plasma levels in intermediate high-risk and low-risk profiles according to a simplified tool based on ESC/ERS Guidelines criteria (ESC/ERS GL Risk), and according to the echocardiographic assessment of right heart (Echo Risk).

When risk was defined according to ESC/ERS Guidelines prognostic criteria, low-risk patients (*n* = 32, 46%) had average Gal-3 levels of 15.9 ± 5.4 ng/mL, intermediate-risk patients (*n* = 31, 44%) had Gal-3 levels of 23.7 ± 7.9 ng/mL, high-risk patients (*n* = 7, 10%) had Gal-3 levels of 24.7 ± 6.8 ng/mL. Due to the inhomogeneous sample size of the three groups, high-risk and intermediate-risk patients were pooled (*n* = 38 patients, Gal-3 levels 24.2 ± 7.5 ng/mL) and compared with low-risk patients. At generalized linear regression analysis with adjustment for etiology, Gal-3 levels were found to be significantly lower in low-risk patients than in intermediate/high-risk profiles (delta = −9.3, 95% CI −12.8 to−5.8, *p* < 0.001; *p* for interaction with etiology 0.146) (Figure 3).

### 3.4. Galectin-3 Levels and Risk Profiles According to the Echocardiographic Assessment of Right Heart

When risk was defined according to the echocardiographic assessment of right heart function, low-risk patients (*n* = 30, 43%) had average Gal-3 levels of 18.5 ± 7.8 ng/mL, intermediate-risk patients (*n* = 36, 51%) had Gal-3 levels of 22 ± 7.5 ng/mL, high-risk patients (*n* = 4, 6%) had Gal-3 levels of 26.3 ± 8.9 ng/mL. Due to the inhomogeneous sample size of the three groups, high-risk and intermediate-risk patients were pooled (*n* = 40 patients, Gal-3 levels 22.1 ± 7.5 ng/mL) and compared with low-risk patients. At generalized linear regression analysis, adjusted for etiology, Gal-3 levels were found to be significantly lower in low-risk profile (delta = −6.3, 95% CI −9.9 to −2.7, *p* = 0.001, without interaction with etiology (*p* for interaction = 0.470) (Figure 3).

## 4. Discussion

The main results of the present study are the demonstration that Gal-3 plasma levels increased linearly in the three risk strata based on the REVEAL 2.0 score and were significantly lower in low-risk patients defined according to the prognostic parameters of ESC/ERS Guidelines. Additionally, Gal-3 levels were associated with a comprehensive echocardiographic assessment of right heart performance.

Circulating biomarkers are investigated in PAH as non-invasive and potentially objective measures for diagnosis, prognosis, and response to therapy, thus overcoming limitations of the clinical and hemodynamic parameters known to be associated with prognosis in PAH (i.e., invasivity, lack of standardization, poor reproducibility). BNP and NT-pro-BNP have been studied most extensively, whereas fewer data have been reported in the literature on the prognostic role of troponin T [14].

Both natriuretic peptides are elevated in many types of pulmonary hypertension; their plasma levels correlate with hemodynamic parameters and change in association with long-term hemodynamic variations, which would suggest a role as a marker of response to PAH-specific treatment [15,16,17,18]. A potential limit for the use of these biomarkers is that their levels are influenced by demographic characteristics, such as obesity, sex, and age and by renal insufficiency [19]. Finally, NT-proBNP levels seem to be higher in PAH associated with connective tissue disease than in idiopathic PAH despite less severe hemodynamic impairment [20]. In addition to natriuretic peptides, the list of molecules investigated in PAH includes, but is not limited to, biomarkers of myocardial injury (troponins); vascular damage/remodeling (von Willebrand factor, angiopoietin, microparticles, growth differentiation factor-15); inflammation/oxidative stress (interleukins, C-reactive protein); end-organ failure (creatinine, sodium, uric acid) [21]. As a matter of fact, current ESC/ERS Guidelines only recommend the evaluation of BNP or NT-pro-BNP at diagnosis and for longitudinal follow up of PAH patients [1].

Circulating biomarkers of fibrosis are of high potential interest; in fact, as much as in heart failure, right ventricular remodeling in PAH involves not only the cardiomyocytes, but also non-myocyte cells and the extracellular matrix, and Gal-3 is a promoter of inflammation and fibrosis. Gal-3 has been correlated with prognosis in SSc [22]. In this connective tissue disease characterized by progressive fibrosis of various organs, Gal-3 plasma levels are strictly linked to the evolution of the disease: Gal-3 levels have been shown to increase during the course of the disease and has been shown to be clearly associated with signs of the advanced organ sclerosis and laboratory parameters of inflammation, which could partially explain the prognostic power of this biomarker in SSc [22,23,24].

The first result of the present study is the demonstration that Gal-3 plasma levels increased linearly in the five risk strata based on the REVEAL 2.0 risk score, which is a validated, easily applied tool for predicting survival in patients with PAH. This holds true both in idiopathic PAH and in PAH associated with SSc, even though Gal-3 levels were higher in SSc patients than in idiopathic PAH patients. To reinforce this evidence is the observation that higher Gal-3 plasma levels were observed in high-risk patients defined according to the prognostic parameters of ESC/ERS Guidelines. Interestingly, Gal-3 plasma levels were also higher in patients with a more advanced impairment of right heart performance. This echocardiographic stratification tool is based on TAPSE, which quantifies the longitudinal systolic function of the right ventricle, which is one of the main mechanisms of blood ejection from the right ventricle, on the degree of tricuspid regurgitation as TAPSE might be overestimated in the presence of significant tricuspid regurgitation, and on the presence of systemic congestion. This datum would therefore suggest that Gal-3 levels are associated with the remodeling process of the right ventricle in response to the increase in afterload.

### Limitations

The association between risk profiles and plasma levels of Gal-3 cannot be considered a demonstration that Gal-3 is a clinically useful prognostic marker; however, given that such multidimensional risk profiles have been tested and validated for prognostic stratification of PAH patients, the association is a valid and strong proof of concept for a prospective study aimed at evaluating whether Gal-3 may provide additional prognostic information in PAH patients. Future studies are also necessary to test whether a multiple biomarker approach, possibly in combination with echocardiographic data of right ventricular structure and function, could be more appropriate to obtain information on the stage of disease and, therefore, on prognosis.

The results of the present study do not elucidate further whether high Gal-3 levels promote PAH disease progression or occur as a secondary consequence of congestive right ventricular dysfunction. Finally, we were unable to evaluate serial changes in Gal-3 levels over time and correlate such changes with changes in risk profile.

## 5. Conclusions

This pilot study provides evidence that Gal-3 plasma levels are different according to risk profiles in PAH patients. This information could prompt future studies aimed at verifying the prognostic significance of this biomarker of fibrosis, in particular when included in composite, weighted risk algorithms, incorporating all elements already considered important for outcome in PAH patients.

## Figures and Tables

**Table 1:** Patients characteristics by Galectin-3 plasma levels.

	Gal-3 < 19,3 (*n* = 35)	Gal-3 ≥ 19,3 (*n* = 35)	P Value
**Clinical and biochemical data**			
Age (y)	51 ± 19	57 ± 15	0.36
Sex (F)	23 (66%)	25 (71%)	0.79
WHO class III/IV	4 (11%)	18 (51%)	0.002
Aetiology (IPAH/SSc/others)	25/3/7	12/14/9	0.015
6MWD (mt)	413 ± 99	346 ± 99	0.007
BNP (ng/mL)	75 (38–135)	235 (100–450)	0.004
**Echocardiographic parameters**			
RV diameter (mm)	34 ± 7	36 ± 6	0.24
RVEDA (cm^2^)	27 ± 8	28 ± 9	0.65
FAC (%)	32 ± 9	30 ± 8	0.35
TAPSE (mm)	20 ± 5	18 ± 3	0.03
LVEI-d	1.2 ± 0.3	1.4 ± 0.2	0.09
RA area (cmq)	22 ± 78	23 ± 6	0.74
Systolic PAP (mmHg)	65 ± 25	77 ± 18	0.02
TR moderate-severe (n, %)	10 (29%)	15 (43%)	0.06
Pericardic effusion (n, %)	8 (23%)	9 (26%)	0.37
IVC plethora (n, %)	5 (14%)	7 (20%)	0.06
**Hemodynamic parameters**			
CI (L/min/m^2^)	2.8 ± 0.6	2.4 ± 0.6	0.009
PAWP (mmHg)	10 ± 3	10 ± 3	0.43
Systolic PAP (mmHg)	70 ± 22	81 ± 24	0.03
Mean PAP (mmHg)	42 ± 13	48 ± 15	0.06
PVR (WU)	6.8 ± 5	10.3 ± 5	0.02
RAP (mmHg)	7.1 ± 3	8 ± 5	0.43
PCa (mL/mmHg)	2± 1.1	1.1 ± 0.5	0.001

Legenda: IPAH = idiopathic pulmonary arterial hypertension. SSc = systemic sclerosis. 6MWT = 6 min walking test. BNP = brain natriuretic peptide. RV = right ventricular. RVED = right ventricular end diastolic diameter. RVEDA = right ventricle end-diastolic area. FAC = fractional area change. TAPSE = tricuspid annular plane systolic excursion. LVEI-d = left ventricular eccentricity index in diastole. RA = right atrium. PAP = pulmonary artery pressure. TR = tricuspid regurgitation. IVC = inferior vena cava. HR = heart rate. CI = cardiac index. PAWP = pulmonary artery wedge pressure. RAP = right atrial pressure. PVR = pulmonary vascular resistance. PCa = pulmonary artery compliance.
